# On the Stiffness of Gold at the Nanoscale

**DOI:** 10.1021/acsnano.1c06947

**Published:** 2021-10-20

**Authors:** Camino Martín-Sánchez, Ana Sánchez-Iglesias, José Antonio Barreda-Argüeso, Alain Polian, Jean-Paul Itié, Javier Pérez, Paul Mulvaney, Luis M. Liz-Marzán, Fernando Rodríguez

**Affiliations:** † MALTA Consolider, DCITIMAC, Facultad de Ciencias, University of Cantabria, Av. Los Castros 48, Santander, 39005, Spain; ‡ CIC biomaGUNE, Basque Research and Technology Alliance (BRTA), Paseo de Miramón 194, Donostia-San Sebastián, 20014, Spain; § Synchrotron SOLEIL, L’Orme des Merisiers St. Aubin, BP48, 91192 Gif-sur-Yvette, France; ∥ IMPMC, Sorbonne Université and CNRS, 4 Place Jussieu, 75005 Paris, France; ⊥ ARC Centre of Excellence in Exciton Science, School of Chemistry, University of Melbourne, Victoria, 3010, Australia; # Ikerbasque, Basque Foundation for Science, Bilbao, 43018, Spain; ∇ Centro de Investigación Biomédica en Red, Bioingeniería, Biomateriales y Nanomedicina (CIBER-BBN), Paseo de Miramón 194, Donostia-San Sebastián, 20014, Spain

**Keywords:** gold nanoparticles, hydrostatic pressure, nonhydrostatic effects, X-ray diffraction, small-angle X-ray scattering, specific volume at nanoscale, bulk modulus

## Abstract

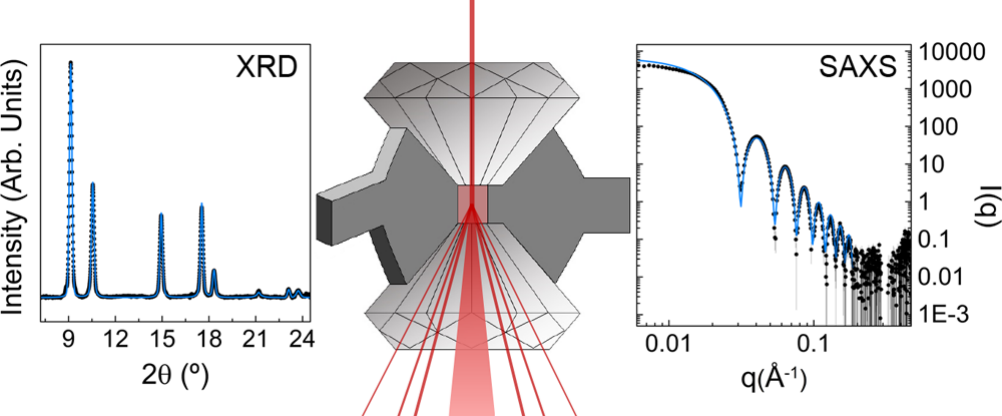

The density and compressibility of nanoscale gold (both nanospheres and nanorods) and microscale gold (bulk) were simultaneously studied by X-ray diffraction with synchrotron radiation up to 30 GPa. Colloidal stability (aggregation state and nanoparticle shape and size) in both hydrostatic and nonhydrostatic regions was monitored by small-angle X-ray scattering. We demonstrate that nonhydrostatic effects due to solvent solidification had a negligible influence on the stability of the nanoparticles. Conversely, nonhydrostatic effects produced axial stresses on the nanoparticle up to a factor 10× higher than those on the bulk metal. Working under hydrostatic conditions (liquid solution), we determined the equation of state of individual nanoparticles. From the values of the lattice parameter and bulk modulus, we found that gold nanoparticles are slightly denser (0.3%) and stiffer (2%) than bulk gold: *V*_0_ = 67.65(3) Å^3^, *K*_0_ = 170(3)GPa, at zero pressure.

## Introduction

The mechanical properties of metallic nanoparticles (NPs) have been the subject of intense research because of their potential technological importance in optical sensing^1,2^ and probe microscopy,^3^ but it remains difficult to obtain accurate theoretical and experimental information at the nanoscale.

The determination of the elastic compliance and related parameters such as Young’s modulus and isothermal bulk modulus (or compressibility), and the critical yield stress, is crucial if we are to understand key mechanical properties, but to date, literature reports have been inconsistent.^4−6^ Although various approaches have been utilized to measure mechanical properties—electron microscopy under tensile stress in single metal nanowires,^1,7,8^ pressure-induced strain by coherent diffraction imaging,^9^ stress induced structural transformations,^10,11^ acoustic methods,^12^ cavitation vibrational frequencies of nanorods,^6,13^ or the compressibility determined by high-pressure X-ray diffraction (XRD)^14−18^—identifying a methodology to unambiguously determine such parameters is still under discussion. Some theoretical models predict that most materials possess superior mechanical strength at the nanoscale because they lack large-scale crystal defects such as grain boundaries and dislocations.^19,20^ Experimentally, the bulk modulus of different materials has been reported to be either independent of NP size,^21,22^ enhanced,^4,23,24^ or reduced,^25,26^ when decreasing the particle size. A recent review on the behavior of NPs under high pressure conditions can be found elsewhere.^27^ It is worth mentioning that the bulk modulus of gold has been determined from *V*(*P*) data obtained by high-pressure XRD, through its equation of state (EOS) for both bulk, micrometer-sized powder and for gold nanoparticles (AuNP) of different sizes and shapes.^2,4,14,18^ Although small differences were found in the reported values of the pressure derivative of the bulk modulus of pure bulk gold (*K*_0_^′^ ∼ 5.3–6), all of them agreed on an isothermal bulk modulus around *K*_0_ = 167 GPa.^18^ Values of *K*_0_ = 167 GPa and *K*_0_^′^ = 5.5(1) were obtained by fitting the *V*(*P*) data measured over the widest hydrostatic pressure range, using helium as the pressure transmitting medium (PTM) to a Vinet EOS.^18^ However, previous XRD studies on bulk Au at high pressure using methanol-ethanol (MeOH-EtOH) 4:1 as PTM reported EOS parameters of *K*_0_ = 167 GPa and *K*_0_^′^ = 5.72.^14^ Even though there is a difference in the reported values of *K*_0_^′^, this uncertainty yields discrepancies in the EOS for determining *P* from *V* below 1% at 30 GPa. Furthermore, this *K*_0_ value agrees within 4% with the bulk modulus determined from the elastic compliances of gold determined from acoustic measurements, such as the ultrasonic pulse-echo technique.^28−30^ However, studies of the EOS of gold at the nanoscale yielded values of *K*_0_ varying by up to 70%. For example, Gu *et al.*(4) reported *K*_0_ = 290(8) GPa (using a MeOH-EtOH 4:1 mixture as the PTM; measurements up to 30 GPa), whereas Hong *et al.*(5) obtained *K*_0_ = 196(3) GPa (using argon as a PTM; measurements up to 71 GPa) for gold nanospheres (AuNS) of similar size. Conversely, the study of extensional and breathing modes in gold nanorods (AuNR) by Hu *et al.*(6) yielded a Young’s modulus, *E* = 64(8) GPa, hence a bulk modulus of *K*_0_ = *E*/3(1–2*v*) = 133 GPa, using a Poisson ratio of *v* = 0.42,^31^ about 19% smaller than that for bulk gold at room temperature.

This data variation may be, to a great extent, due to technical limitations at the nanoscale and to sample heterogeneity (polydispersity and aggregation). These effects are enhanced in high-pressure experiments dealing with concentrated nanoparticle dispersions or compacted samples. In such cases, nanoparticles experience nonhydrostatic conditions, giving rise to additional axial stress components and causing their XRD patterns to deviate from those measured under hydrostatic conditions. As pointed out in previous studies,^17,18,32^ the lattice parameters obtained under nonhydrostatic conditions can deviate significantly from those obtained under hydrostatic conditions, thus giving rise to different bulk moduli depending on both pressure conditions and diffraction geometry.^23^ Furthermore, as we show in this work, the lack of hydrostaticity at the nanoscale enhances the stress fields acting on the nanoparticle—these may be up to a factor of 10 higher than those acting on the bulk phase—thus emphasizing the variability of the EOS derived from XRD measurements under nonhydrostatic conditions.

We present herein a high-pressure XRD study, using a dilute AuNP solution itself as the PTM acting on individual—nonaggregated—AuNS and AuNR. Compacted gold powder of 2 μm average grain size was used as a pressure calibrant in each load, enabling us to simultaneously compare both bulk and nanoscale gold XRD patterns under the same environmental—pressure and temperature—conditions. We aimed at elucidating whether gold nanoparticles are stiffer than bulk gold, as well as their mechanical stability under severe nonhydrostatic conditions, and whether the shape and size of the AuNP remain constant after pressure release.

Our methodology was based on using dilute NP dispersions (<10^12^–10^14^ AuNP/cm^3^; [Au] = 5–13 mM), coated with polyethylene glycol (PEG) to guarantee dispersion of individual NPs in a MeOH-EtOH 4:1 mixture, which is the liquid PTM with the highest hydrostatic range.^33^ This solvent allows us to reach a hydrostatic limit up to 11 GPa—about 2 GPa less when containing dispersed AuNPs^34^—and therefore a relatively wide pressure range for the precise determination of *V*(*P*) with Δ*V*/*V* ∼ 10^–4^. The AuNP shape, size distribution, and monodispersity were determined by transmission electron microscopy and UV–vis spectroscopy (see Experimental Methods). The *in situ* control of the AuNP dispersion under high-pressure conditions within a diamond anvil cell (DAC)—the device used to produce high-pressure conditions on the NP—was probed by small-angle X-ray scattering (SAXS), which is highly sensitive to the NP aggregation state (through the structure factor) as well as to the NP shape and size (through the form factor).^35^ Although the use of SAXS in high-pressure experiments with DAC was restricted to 5 GPa due to technical limitations imposed by the X-ray beam spot size (about 200 μm), thus requiring an 800 μm culet diamond and 0.5 mm thick anvils, this technique can still provide crucial *in situ* information on the NP dispersion under high-pressure conditions, which has not been exploited so far for this purpose.

Nonhydrostatic effects on both XRD and SAXS measurements were also investigated through the axial-stress-averaged model, which allowed us to separate the hydrostatic stress from the biaxial stress components obtained in a DAC.^36^ We demonstrate that the model accounts well for Bragg angle deviations with respect to a simple cubic lattice analysis. However, due to the biaxial stress distribution around randomly oriented AuNPs, we find that the resultant lattice volume is not sufficiently precise for determining a suitable EOS for gold in the nonhydrostatic pressure range. This result can explain the widespread values of Young’s modulus and isothermal bulk modulus, reported in previous XRD studies.^4−6^ Our methodology additionally provides an adequate description of the stress distribution within the NP. We used TEM to explore NP deformation or alloying, by recovering the sample after pressure release.

## Results and Discusion

### X-ray Diffraction by Gold Nanoparticles at High Pressure

**Figure 1:**
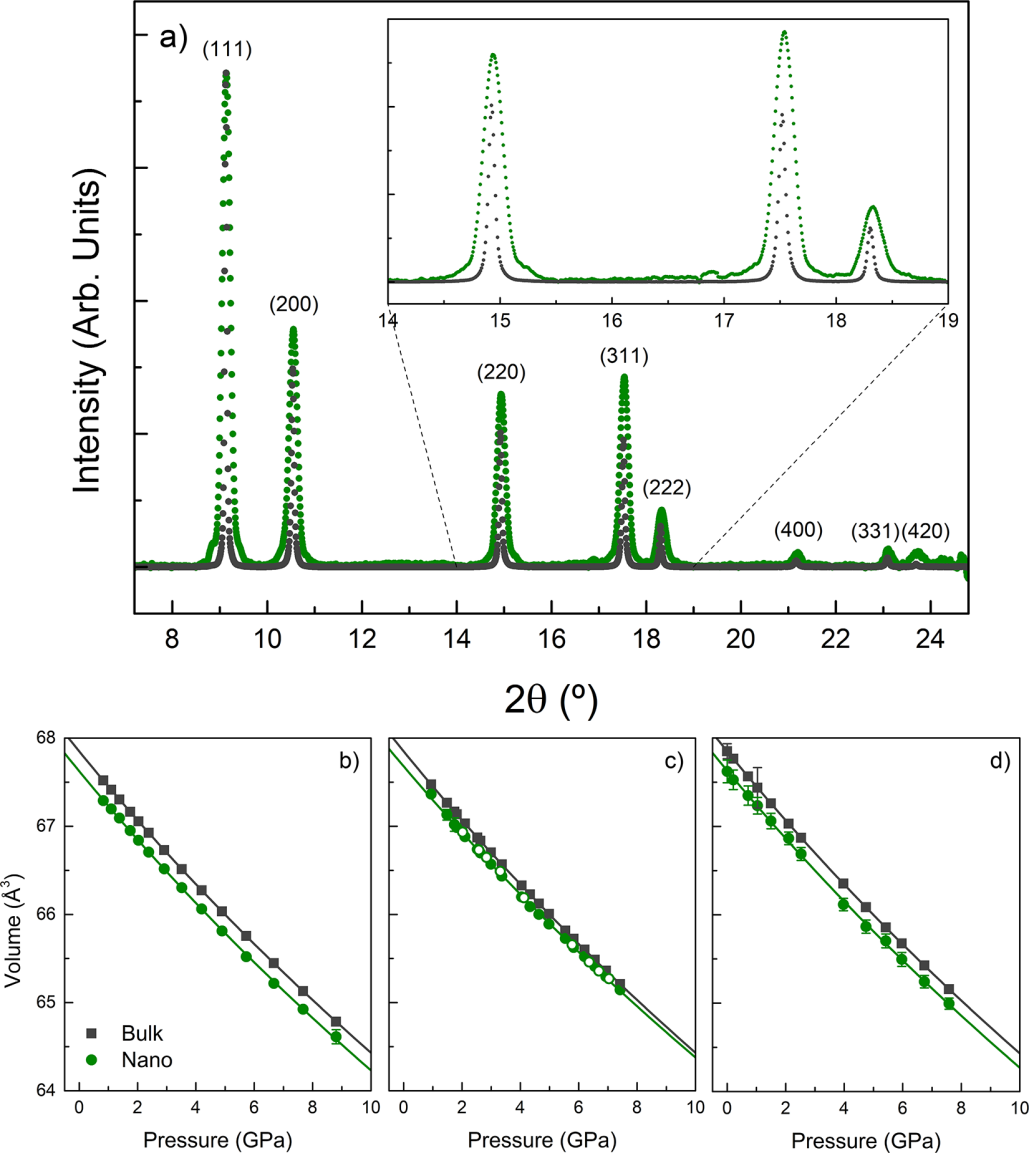
X-ray diffraction of AuNP at high hydrostatic pressure. (a) Diffraction patterns of 12.3 nm AuNS colloids in MeOH-EtOH 4:1 (green) and Au micrometric powder (gray), both at 1.1 GPa. Note the broadening and shift toward higher Bragg angles of the NP diffraction peaks, with respect to those of bulk gold. The inset shows a magnification of the (220), (331), and (222) peaks. Intensities were normalized to the (111) reflection. Plots (b)–(d) show the pressure dependence of the fcc cell volume for 12.3 nm diameter AuNS, 28.2 nm diameter AuNS, and 10.7 nm diameter AuNR with AR = 3.4, respectively. Filled symbols correspond to experimental data; solid lines correspond to fits of the Vinet EOS to the measured *V*(*P*) data. Empty circles in (c) correspond to experimental points in downstroke, obtained during the reversibility test on the 28.2 nm AuNS solution in the hydrostatic range. Error bars in volume are either indicated or smaller than the symbols.

Figure 1 shows typical XRD patterns of 12 nm AuNS and Au micrometric powder, acquired under the same pressure conditions and environmental setup. The XRD patterns at 1.1 GPa show two important differences: (1) Bragg peaks in AuNS are broader than in bulk gold, and (2) the lattice parameter of AuNS (*a* = 4.0655 ± 0.0005 Å) is slightly smaller than that of bulk Au (*a* = 4.0699 ± 0.0005 Å); *a*_0_ = 4.0741 Å and 4.0787 (±0.0002) Å for 12 nm AuNS and bulk Au at ambient pressure, respectively, meaning that the NPs are *ca*. 0.3% denser than the bulk. These slight differences in lattice parameter and density were found to be maintained within the whole hydrostatic pressure range.

Although there is a clear difference in the values of the lattice parameters obtained for nanosized and bulk gold at zero pressure, the variations with pressure in the hydrostatic regime are very similar. Figure 1b–d shows the fit of the experimental data volume to a Vinet EOS^37^
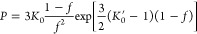
1with *f* = (*V*/*V*_0_)^1/3^ (= *a*/*a*_0_ in cubic systems). The obtained values of the bulk modulus and the volume at zero pressure for the three investigated samples are given in Table 1. We used the same value of *K*_0_^′^ = 5.72 reported by Heinz *et al.*(14) in the fits for all three samples. This left two fitting parameters, to avoid parameter uncertainty and to allow comparison of bulk modulus and volume at zero pressure among all three AuNP samples and bulk Au. Within the fitting accuracy, the same *V*_0_ and *K*_0_ values were obtained, using either a Vinet or a third-order Murnaghan EOS^38^ for each sample.

**Table 1:** Fitting Parameters Using a Vinet Equation of State^a^

	*K*_0_ (GPa)	*V*_0_ (Å^3^)	*a* (Å)
12.3 nm AuNS	167.8(9)	67.625(9)	4.0741(2)
28.2 nm AuNS	173.4(1.1)	67.680(9)	4.0752(2)
AR = 3.4 AuNR	169.7(1.4)	67.631(13)	4.0743(3)
Au Bulk^14^	167	67.852(9)	4.0787(2)

a The first derivative of the bulk modulus was fixed at *K*_0_^′^ = 5.72.^14^ Fit errors are given in parentheses.

Contrary to previously reported results,^4,5^ we found a compressibility for the NP that was slightly lower (but very similar) to that of bulk gold for all the samples studied. Comparing the results of the three samples (12 and 28 nm AuNS and 10.7 nm diameter with *AR* = 3.4 AuNR) having an average value of the bulk modulus, *K*_0_ = 170(3) GPa, we consistently observed a small difference, suggesting that AuNP are less compressible than bulk Au, even though we found the bulk moduli to be, within experimental uncertainty, very similar to each other. Nevertheless, it must be noted that, for each experiment, a AuNP sample and a sample of bulk gold were loaded together into the hydrostatic cavity. In each case, the bulk modulus derived through the EOS (eq 1) was systematically higher for the nanoscale sample: *K*_AuNP_ > *K*_bulkAu_. By comparing the XRD patterns of AuNP and bulk Au, we found that the fcc cell volume at zero pressure for gold nanoparticles was 0.3% smaller than that of the bulk metal, the average value being *V*_0_ = 67.65(3) Å^3^ for AuNP and *V*_0_ = 67.852(9) Å^3^ for bulk Au. Interestingly, we found that the fcc cell volume at zero pressure decreases with decreasing NP volume (see Figure S1 in the Supporting Information (SI)). This behavior agrees with previously published data for gold and silver NPs.^4,39−41^ It should be noted that the volume changes were completely reversible, during both upstroke and downstroke, under hydrostatic conditions (see Figure 1c and Figure S2 in the SI).

**Figure 2:**
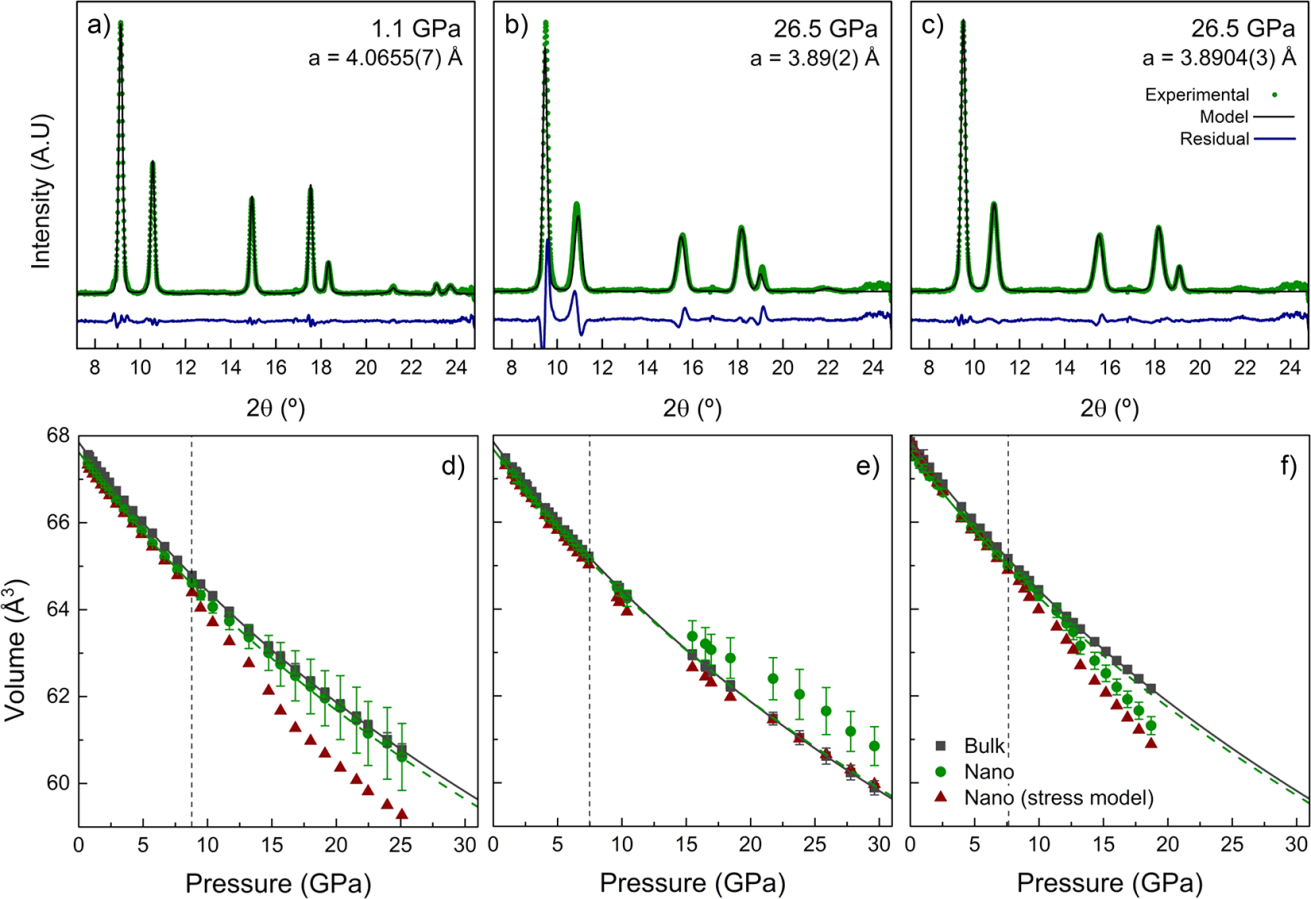
X-ray diffraction under nonhydrostatic conditions. XRD patterns for selected pressures in the hydrostatic and nonhydrostatic pressure ranges, for 12.3 nm AuNS. XRD patterns in (a) and (b) were modeled within a stress-free cubic system, whereas the pattern in (c) was modeled considering the stress state of the system.^36^ Note the abrupt deviation of the stress-free model in the nonhydrostatic region in the XRD pattern in (b). Plots (d)–(f) show the pressure dependence of the fcc cell volume for 12.3 nm AuNS, 28.2 nm AuNS, and 36.4 × 10.7 nm^2^ (*AR* = 3.4) AuNR, respectively. Gray squares correspond to experimental data for bulk gold; green circles correspond to nanosized gold, assuming a stress-free cubic model system; and dark red triangles represent nanosized gold, considering stress by following a model reported elsewhere.^36^ Solid lines correspond to fits using the Vinet EOS; dashed lines correspond to the extrapolation of the hydrostatic EOS. Error bars in volume are indicated or are smaller than the symbols. The vertical dashed lines show the hydrostatic limit for the PTM.

### Nonhydrostatic Effects

Figure 2 illustrates the effects of nonhydrostaticity on the XRD patterns. Once the pressure transmitting medium solidifies, there is a clear deviation from the hydrostatic behavior: Bragg peaks broaden markedly and modeling of the diffraction pattern from a single lattice parameter ⟨*a*⟩ (cubic system without strain) is inadequate, as the sample undergoes a tetragonal-like-distortion due to the appearance of uniaxial stresses along the DAC loading direction (see Figure 2b). In order to properly describe the XRD patterns under nonhydrostatic conditions, it was necessary to model the stress field of the solidified solution following the stress model reported in ref (36) (see details in the SI). However, although the model accounts for the measured XRD pattern in the nonhydrostatic range fairly well, it provides inconsistent results for the real *V*(*P*) data when we consider the “hydrostatic” lattice parameter extracted from the model (Figure 2). The *V*(*P*) values derived from the stress model in our experiments are systematically lower than those obtained from a stress-free cubic system. This behavior, which is associated with the diffraction geometry of the experiment with the X-ray **k** vector along the DAC axis, *i.e.*, axial stress direction, has also been observed in other systems.^36^

Consequently, the bulk moduli derived by fitting the *V*(*P*) data to a Vinet EOS over the whole pressure range are incongruent: 171(1) GPa for 12.3 nm AuNS, 219(4) GPa for 28.2 nm AuNS, and 146(4) GPa for AuNR. These results clearly illustrate that, even when working with the same type of colloidal AuNP samples and under the same experimental conditions, different values of the bulk modulus can be obtained depending on the stress field. This result reaffirms the importance of working under hydrostatic conditions to obtain correct values from the EOS. It thus turns out that application of a hydrostatic pressure to a compacted, polycrystalline nanoparticle necessarily leads to internal stresses within the nanoparticles, which can modify the XRD pattern with respect to the unstressed NP. This situation also arises in highly concentrated NP solutions, where pressure can induce aggregation or alloying phenomena. In this respect, Gu *et al.*(4) worked with AuNP powder covering 70% of the total volume of the sample chamber (MeOH-EtOH 4:1 as PTM 30%). The presence of nonhydrostatic stresses in the samples due to bridging between the anvils is likely, even when working with a truly hydrostatic PTM, and this probably explains the large bulk modulus of 290(8) GPa that was obtained. Therefore, working with dilute colloidal dispersions is crucial to guarantee the application of homogeneous stresses to individual nanoparticles.

**Figure 3:**
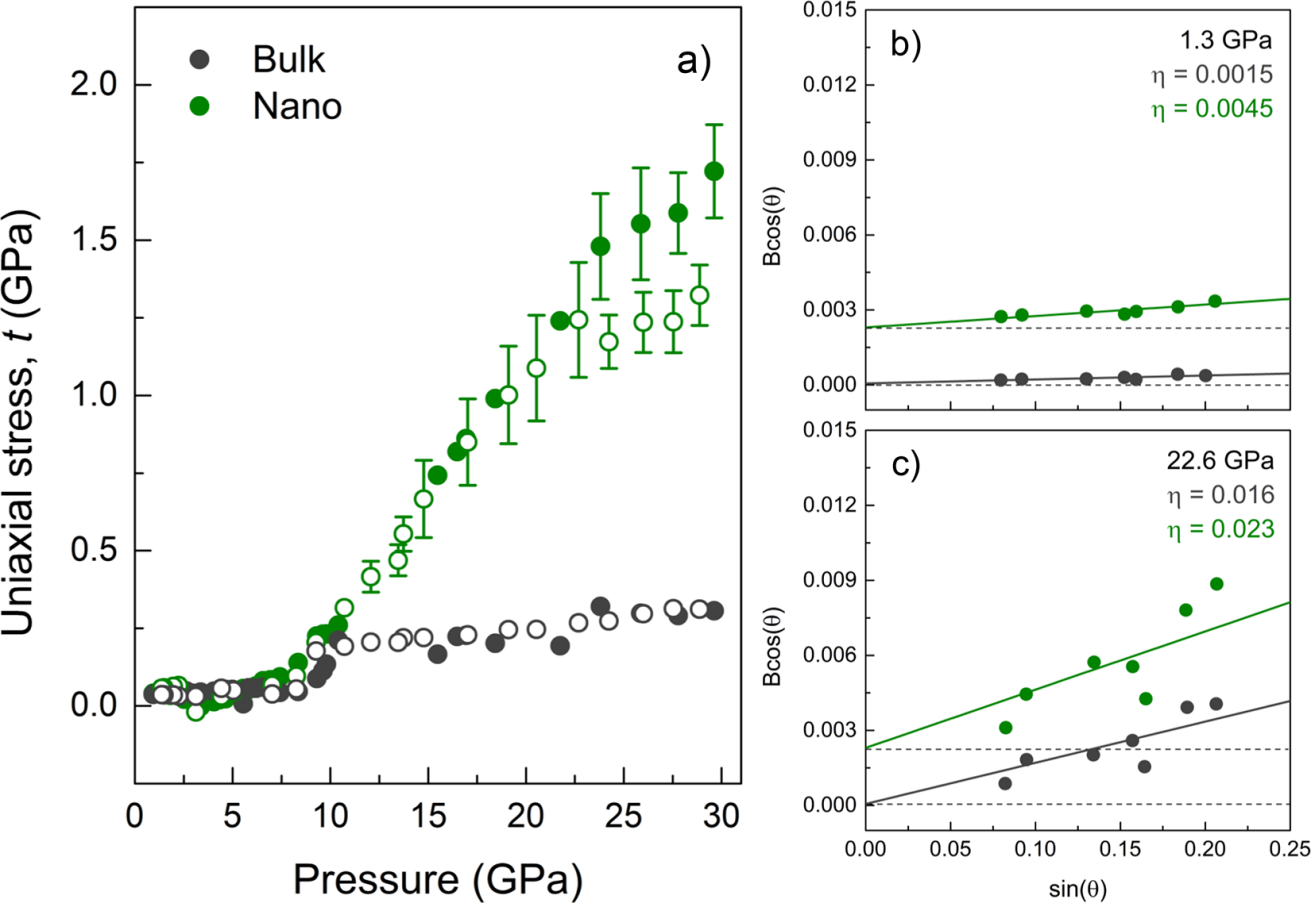
Uniaxial stress in AuNP at high pressure. (a) Uniaxial stress component *t* = (*t*_11_ – *t*_33_) as a function of pressure, for 2 μm gold powder (gray) and 28.2 nm gold nanospheres (green). Empty circles correspond to the experimental points in downstroke. (b, c) Williamson–Hall plots for selected pressures in the hydrostatic (b) and nonhydrostatic (c) pressure ranges. Note the different *y*-intercepts of the curves for the nanosized and bulk samples, derived from the different crystallite sizes and the strong increase in slope, *i.e.*, strain η, under nonhydrostatic conditions.

Although the stress model does not provide precise values of the lattice parameter, it does give information on the axial stress field acting on the NP, which in turn allows us to precisely identify the solidification pressure of the PTM and the increase in axial stress with pressure (Figure 3a). The axial stress, which was derived from the Bragg peak shifts of the measured XRD patterns, was consistent with the NP strain derived from the Williamson–Hall (WH) plots^42^ (Figure 3b,c), relating XRD peak broadening to the combined effects of crystallite size and lattice strain (see Figure S4 in the SI). Interestingly, while the uniaxial stress reached values of 0.2–0.3 GPa for bulk Au in the studied pressure range, it reached 2 at 30 GPa in AuNP. This means that the axial stress acting on the NP was enhanced by an order of magnitude with respect to that on bulk Au, even though both samples were measured under the same environmental conditions in the DAC.

### Small-Angle X-ray Scattering by Gold Colloids at High Pressure

**Figure 4:**
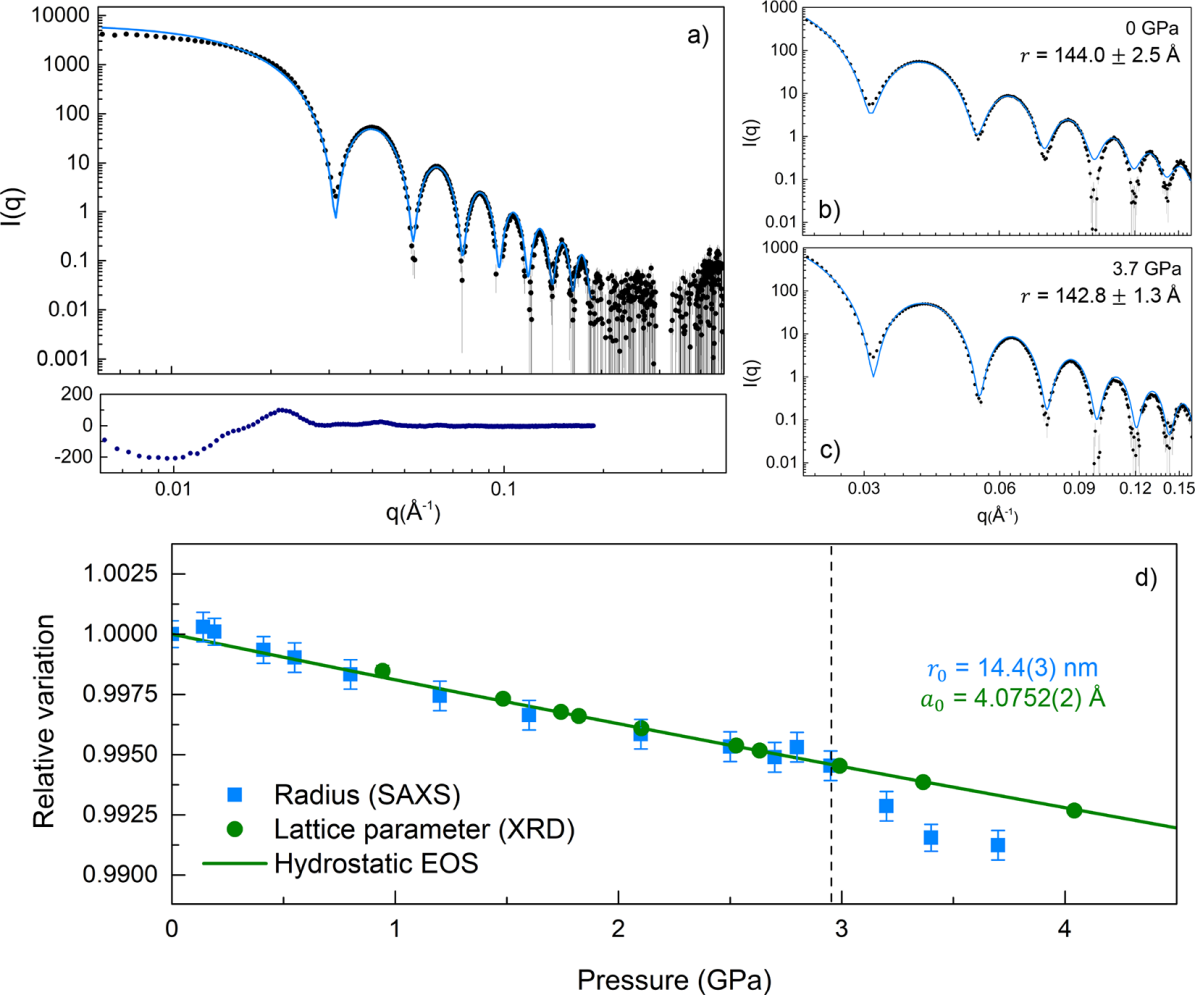
Small-angle X-ray scattering at high pressure. (a) SAXS *I*(*q*) pattern from 28.2 nm AuNS solution in ethanol, recorded right after PTM solidification at 2.9 GPa. Filled circles correspond to experimental data, the solid blue line represents the calculated *I*(*q*) curve for a monodisperse (individual) NP structure factor. The fit residuum is shown below. (b, c) Magnification of the SAXS *I*(*q*) pattern corresponding to the form factor of a sphere for two selected pressures: 0 GPa (hydrostatic) and 3.7 GPa (nonhydrostatic). (d) Pressure-dependence of the relative variation of the 28.2 nm AuNS radius and lattice parameter, measured by SAXS and XRD, respectively. The solid line represents the corresponding Vinet EOS derived from the hydrostatic pressure region (Table 1). Error bars in the relative variation of the lattice parameter are smaller than the symbols. The vertical dashed line shows the hydrostaticity limit of the PTM.

The analysis of the XRD data was carried out on the assumption that AuNP solutions remained fully dispersed over the whole pressure range, *i.e.*, that the PTM thus acted on individual nanoparticles. This assumption is crucial, as pressure-induced NP aggregation or alloying may itself induce axial stress components, and in turn modifications to the cubic XRD pattern, leading to an erratic determination of *V*(*P*). Given the current technical difficulties to perform electron microscopy or individual NP imaging (for NP sizes of about 30 nm)^9^ in a DAC for *in situ* control of the NP solution, we exploited the potential of SAXS to analyze the state of aggregation, as well as the shape and size of NPs, as a function of pressure. Figure 4 shows representative SAXS patterns for 28 nm AuNS in EtOH solutions, together with the corresponding simulations, considering a Gaussian size distribution and the absence of a structure factor (no interactions between individual NPs). The *I*(*q*) analysis confirms monodispersity of the (nonaggregated individual particles) AuNS solution (Figure 4a), and the spherical shape, with an average sphere radius *r* = 14.4 nm and a standard deviation of 0.3 nm, a value fully consistent with the mean NP diameter determined by TEM, *d* = 28.2 ± 0.4 nm (Figure 4b). Notably, the AuNS solution remained colloidally stable over the whole analyzed pressure range during both upstroke and downstroke. No evidence of aggregation was observed in any of the studied solutions, even under nonhydrostatic pressure conditions, as is evident from inspection of Figure 4a). In fact, the residual plot indicates the existence of a repulsive force between nanospheres, likely derived from the stabilizing agent (PEG) adsorbed onto the NP surface, which contributed to the observed colloidal stability. We compare in Figure 4d the relative variation of the SAXS-determined radii (radius *r*_0_ = 14.4 nm) and the corresponding lattice parameter measured by XRD (*a*_0_ = 4.0752(2) Å, determined from the AuNS EOS data collected in Table 1). Interestingly, relative variations in both lattice parameter and sphere radius decrease at the same rate with increasing pressure; *i.e.*, *r* and *a* are proportional to each other. Although the data derived from SAXS have a lower precision and, due to the experimental conditions, the accessible hydrostatic pressure range is also lower, both techniques yield fully consistent results. Similar results were additionally obtained for AuNR dispersions (see Figures S7 and S8 in the SI). These results reveal that the AuNPs do not deform during pressure treatment: the critical shear stress for plastic deformation is not reached under the high-pressure conditions in our experiments. TEM analysis of recovered AuNSs from the colloid after a high-pressure treatment of 31 GPa in MeOH-EtOH 4:1 shows no evidence of significant plastic deformation (1 out of 300 deformed NPs), the size distribution being identical before and after the pressure treatment (see more details in the SI). The recovered AuNR colloid shows the same size and shape distribution after compression to 10 GPa in the hydrostatic regime, but plastic deformations were identified under strong nonhydrostatic conditions at 20 GPa.

## Conclusions

We have demonstrated that the compressibility of gold at the nanoscale (nanospheres of 12.3 and 28.2 nm and nanorods of 36.4 × 10.7 nm^2^) is slightly lower than that of the bulk metal (2 μm grain size powder). The corresponding EOS was determined for each material from precise *V*(*P*) data obtained by XRD from individual AuNP and gold powder, under identical pressure conditions and environmental setup, using synchrotron radiation. These experimental conditions enabled us to carry out a precise comparison of bulk and nanoscale gold metal, and to extract reliable values of their respective volumes and bulk moduli under hydrostatic conditions. We showed that gold at the nanoscale is slightly denser (0.3%) and stiffer (2%) than bulk gold, with *V*_0_ = 67.65(3) Å^3^ (*a*_0_ = 4.0745(6) Å) and bulk modulus *K*_0_ = 170(3) GPa, obtained as an average of the values obtained for all NP solutions. This result is consistent with the EOS of gold because the reduction in volume at the nanoscale (−0.3%) corresponds to an *effective* Laplace pressure in the AuNP of 0.5 GPa. This corresponds to a bulk modulus increase of 3.0 GPa according to the pressure derivative of the bulk modulus of *K*′ = 5.72, which, in turn, implies a bulk modulus increase of 1.8% in the nanoscale metal, consistent with the measured bulk moduli at the nanoscale.

We confirmed that the emergence of uniaxial stresses after solidification of the pressure transmitting medium—the AuNP solution itself—induced a deviating behavior of the XRD pattern from an ideal cubic system; the higher the nonhydrostatic pressure, the higher the axial stress, and the larger the shift of Bragg reflections. We interpreted XRD shifts according to the stress state model, which provides a good description of the axial stress acting on the AuNP, compatible with the additional broadening of XRD Bragg peaks in the nonhydrostatic pressure range. We showed that the effect of axial stress on the diffraction peaks was enhanced in NPs as compared to bulk gold, thus revealing an axial stress at the NPs, which is an order of magnitude higher than in the bulk phase under the same nonhydrostatic pressure conditions. However, the hydrostatic volume *V*(*P*) of AuNPs derived in the nonhydrostatic range underestimates the real anisotropic volume *V*(*P*), thus yielding erroneous estimates of the EOS at the nanoscale. This work highlights the importance of working under hydrostatic conditions to extract precise values of *V*_0_ and *K*_0_ which are to be compared with those of bulk gold. We have also demonstrated the suitability of SAXS to probe the aggregation state of AuNP under high-pressure conditions of up to 5 GPa, as well as their shape and size. Finally, we conclude that the AuNP colloidal solutions maintain their stability within the whole analyzed pressure range, and the relative variation of SAXS-determined AuNS radius and corresponding XRD-lattice parameters fairly agree in the accessible pressure range. TEM images of the recovered AuNP solution showed that the shape and size distribution of the nanoparticles are, within experimental accuracy, unchanged before and after applying pressure.

## Experimental Methods

### NP Synthesis

Chemicals: Gold(III) chloride trihydrate (HAuCl_4_·3H_2_O, ≥ 99%), hexadecyltrimethylammonium bromide (CTAB, ≥ 99%), sodium borohydride (NaBH_4_), hexadecyltrimethylammonium chloride (CTAC, 25 wt % in water), benzyldimethylhexadecylammonium chloride (BDAC), ascorbic acid (AA, ≥ 99%), hydroquinone (HQ, ≥ 99%), silver nitrate (AgNO_3_, ≥ 98%), *O*-[2-(3-mercaptopropionylamino)ethyl]-*O*′-methylpolyethylene glycol (PEG-SH, Mw: 5K) were purchased from Sigma-Aldrich. Ethanol and methanol were purchased from Scharlab. All chemicals were used without further purification. Milli-Q water (resistivity 18.2 MΩ·cm at 25 °C) was used in all experiments. All glassware was cleaned with aqua regia, rinsed with Milli-Q water, and dried before use.

Synthesis of single-crystalline AuNS and AuNR: Single-crystalline AuNS and AuNR were synthesized *via* well-established seeded-growth methods.^43,44^ First, gold seeds (∼1.5 nm) were prepared by fast reduction of HAuCl_4_ (5 mL, 0.25 mM) with freshly prepared NaBH_4_ (0.3 mL, 10 mM) in aqueous CTAB solution (100 mM) under vigorous stirring for 2 min at room temperature and then kept undisturbed at 27 °C for 30 min to ensure complete decomposition of sodium borohydride. The mixture turns from light yellow to brownish, indicating the formation of gold seeds. To grow 12 nm gold nanospheres from gold seeds, an aliquot of seed solution (0.6 mL) was added under vigorous stirring to a growth solution containing CTAC (100 mL, 100 mM), HAuCl_4_ (0.36 mL, 50 mM), and ascorbic acid (0.36 mL, 100 mM). The mixture was left undisturbed for 12 h at 25 °C. The solution containing gold nanoparticles was centrifuged (9000 rpm for 1 h) to remove excess CTAC and ascorbic acid and redispersed in 1 mM CTAB to a final gold concentration of 1 mM.

To grow 12 nm gold nanospheres up to 28 nm in diameter, an aliquot of 12 nm AuNS solution (2.14 mL, 1 mM) was added under magnetic stirring to a growth solution (100 mL) containing benzyldimethylhexadecylammonium chloride (BDAC, 100 mM), HAuCl_4_ (0.25 mM), and ascorbic acid (0.5 mM). The mixture was left undisturbed for 30 min at 30 °C and then washed twice by centrifugation (8000 rpm for 1 h). The particles were finally dispersed in 1 mM CTAB to a final gold concentration equal to 1 mM.

Gold nanorods were synthesized as described elsewhere^44^ with some modifications. Gold nanorods were prepared by adding an aliquot of gold seeds (∼1.5 nm, 1 mL) under vigorous stirring to a growth solution containing CTAB (100 mL, 100 mM), HAuCl_4_ (1 mL, 50 mM), HQ (15 mL, 100 mM), and AgNO_3_ (1.4 mL, 10 mM). The stirring was stopped after 5 min, and the mixture was left undisturbed for 2 h at 30 °C. The nanoparticles were washed by two centrifugation rounds (8000 rpm, 30 min) to remove excess reagents. After the second centrifugation step, the solution was redispersed in CTAB (100 mM) to a final gold concentration of 1 mM. Gold nanorods (15 mL, 1 mM) were partially oxidized with Au^3+^ (3 mL, 1 mM, 1 mL/h) until the longitudinal absorption band was located at 694 nm. Then, the solution was centrifuged (9000 rpm for 1 h) and redispersed in CTAB 1 mM. The concentration of gold for ligand exchange was 1 mM.

Ligand exchange:^45^ To replace the surfactant and transfer the gold nanoparticles to the alcoholic mixture, thiolated polyethylene glycol (PEG-SH) of molecular weight of 5K was used. An aqueous solution of PEG-SH (25.4 mg and 10.9 mg for 12 and 28 nm gold nanospheres, respectively, and 21.3 mg for gold nanorods, dissolved in 2 mL of water) dispersion was added dropwise under stirring to a dispersion of gold nanoparticles (12 mL, 1 mM) in 1 mM CTAB. The solution was left for 2 h under stirring and then centrifuged twice in a mixture of methanol-ethanol (4:1). Pegylated gold nanoparticles were finally dispersed in methanol-ethanol (4:1).

**Figure 5:**
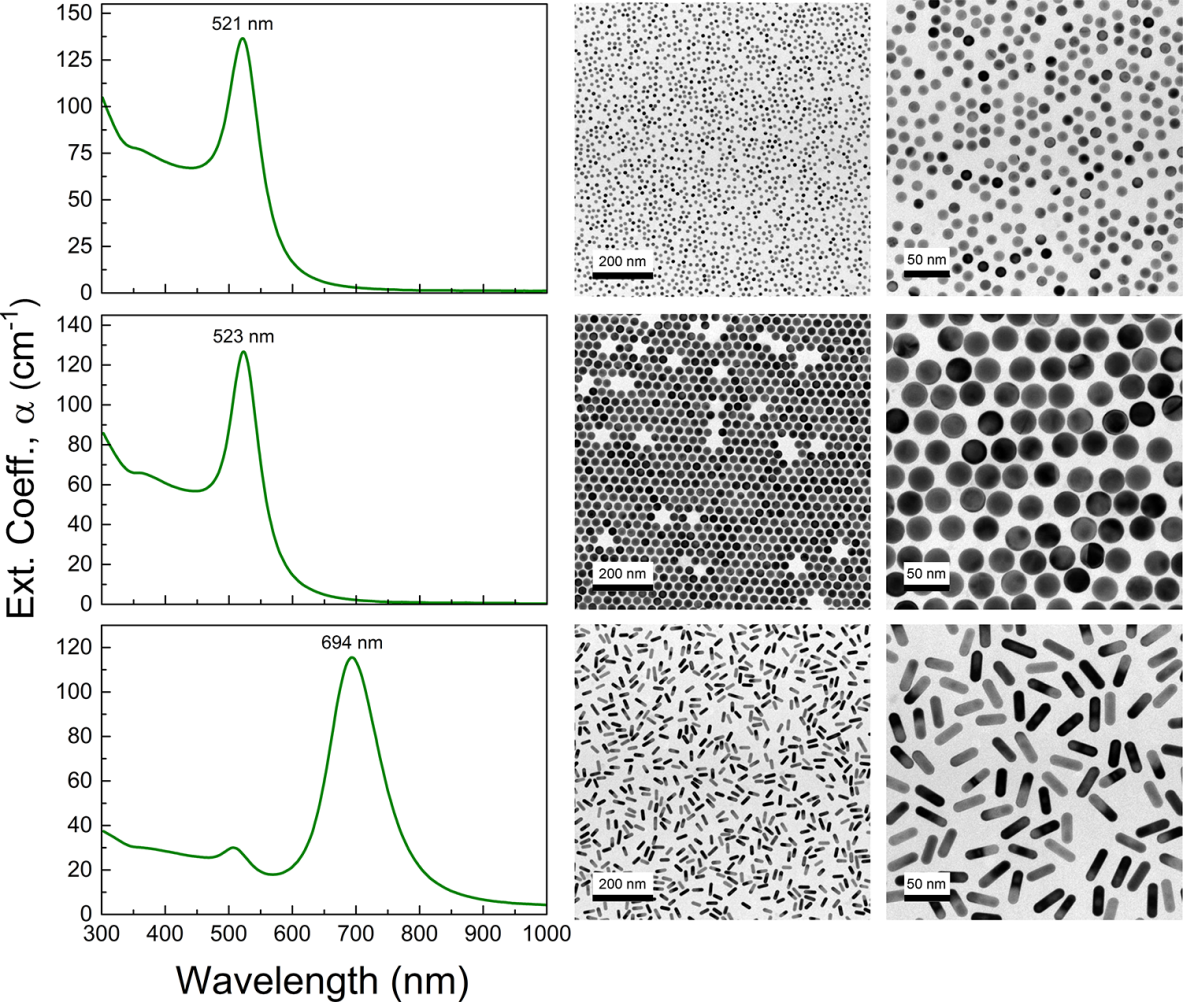
Gold nanoparticle characterization. Optical extinction spectra and representative TEM images at different magnifications of the nanoparticles used in the experiments, 12.3 nm AuNS with [Au] = 12.7 mM, and [NS] = 1.4 × 10^14^ cm^–3^ (top row); 28.2 nm AuNS with [Au] = 11.0 mM, and [NS] = 9.7 × 10^12^ cm^–3^ (middle row); and 10.7 × 36.1 nm^2^; AR = 3.4 AuNR with [Au] = 5.2 mM, and [NR] = 2.3 × 10^13^ cm^–3^ (bottom row).

Representative TEM images and extinction spectra of the AuNP colloids employed in the experiments are shown in Figure 5. The investigated AuNS have average diameters of 12.3 ± 0.3 and 28.2 ± 0.4 nm, and their extinction spectra show characteristic surface plasmon resonance (SPR) bands centered at 521 and 523 nm, respectively. AuNRs have a mean length of 36.1 ± 0.6 nm, a mean diameter of 10.7 ± 0.4 nm, and an AR distribution 3.4 ± 0.2, and the optical spectrum shows the characteristic band structure associated with a transversal SPR at 510 nm and a longitudinal SPR at 694 nm.

### High-Pressure X-ray Diffraction

XRD measurements of AuNP under high pressure conditions were performed at the SOLEIL Synchrotron (France), using the PSICHÉ beamline. 2D XRD data were collected on a CdTe2M Dectris detector, using a monochromatic X-ray beam with a wavelength of 0.3738 Å, focused to a beam size of 12 × 14 μm^2^ (FWHM). A membrane DAC with a 300 μm diameter culets and automatic control of the membrane pressure was employed as the pressure generator. A parallel configuration geometry for diffraction (incident X-ray beam parallel to the DAC load axis) was used. Gold colloids were loaded into a 150 μm diameter hole, within a stainless-steel gasket that had been preindented to a thickness of 35 μm. A compacted polycrystalline gold powder of 2 μm grain average size was used as a pressure calibrant, following the EOS reported by Heinz *et al.*,^14^ using the MeOH-EtOH 4:1 AuNP dispersion itself as the pressure transmitting medium. *V*(*P*) data of the gold calibrant were fitted to a Vinet EOS which yielded *a*_0_ = 4.0787(2) Å; *V*_0_ = 67.852(9) Å^3^; *K*_0_ = 167.0; and *K*_0_^′^ = 5.7(2). We followed the EOS reported by Heinz *et al.*,^14^ since the experimental measurements were carried out using MeOH-EtOH 4:1 as PTM which is similar to our system. However, using this equation instead of the one reported more recently by Dewaele *et al.*(18) using helium as PTM only induces a maximum deviation of 0.3 GPa in 30 GPa, *ca*. 1%. The reversibility of the process was studied within both the hydrostatic regime (0–7 GPa in upstroke and downstroke) and in the nonhydrostatic regime (0–30 GPa in upstroke and downstroke).

The described configuration setup provides suitable XRD patterns covering the (111), (200), (220), (311), and (222) Bragg reflections over the whole pressure range and for all samples. Reflections corresponding to (400), (331), and (420) planes could also be recorded in the low-mid pressure range (0–15 GPa) for 12.3 nm AuNS and for AuNR. The lattice parameter was determined by means of a Le Bail-type analysis,^46^ fitting pseudo-Voigt profiles to the diffraction patterns. Precise lattice parameters (Δ*a*/*a* ∼ 5 × 10^–5^) and FWHM (Δ*FWHM*/*FWHM* ∼ 10^–2^) were obtained in the hydrostatic regime (0–7 GPa range), with a residue *R*_*w*_ of *ca*. 1%. The fitting quality decreased progressively in the nonhydrostatic region (*P* > 9 GPa), with values of *R*_*w*_ around 10% caused by axial stress components. The stress-induced shifts of the Bragg peaks with respect to pure hydrostatic conditions were analyzed by means of the Stress State model reported by Singh,^36^ which allowed us to consider the effects of axial stress at the NP (see more details in the SI). We used the stress continuity across grain boundaries approximation,^47^ as it provides a better description of the stress field in the DAC. Our analysis showed that the Reuss approximation provided the best match to XRD patterns, as compared to a mixed Reuss–Voigt^48^ shear moduli approximation. This model allowed us to obtain those uniaxial stresses that are superimposed on top of the hydrostatic pressure, from the resulting lattice strains derived through the XRD diffraction patterns, and the elastic compliances of bulk gold.^28−30^

### Small-Angle X-ray Scattering at High Pressure

SAXS measurements were performed on the SWING beamline, at the SOLEIL Synchrotron. The beamline was adapted to high-pressure SAXS by inserting a membrane diamond anvil cell equipped with 1 mm thick, 3 mm diameter anvils ground with 0.8 mm culets. This anvil geometry allowed us to work with gasket cavities of 300 μm in diameter, which properly fit onto the 200 × 150 μm^2^ X-ray beam spot and attenuate the beam intensity to only 20% at the working energy. These conditions are crucial for obtaining suitable SAXS signals, *I*(*q*), for structural analysis within the 0–5 GPa pressure range. The experiments were carried out employing a monochromatic X-ray beam of 0.8265 Å wavelength passing through the DAC and focused at the two-dimensional EIGER-4 M detector position, located 2030 mm downstream of the sample. The selected sample-detector distance and beam energy (15 keV) allowed us to locate the optimum scattering angular range, in order to obtain the most precise values of the form factor (size and shape of the NP) and the structure factor (aggregate formation or NP precipitation). The gold colloids were loaded onto a membrane DAC with automatic control over the membrane pressure, 800 μm culet diameter diamonds, and a 300 μm drilled hole in a CuBe gasket, pre-indented to 100 μm. Ruby microspheres of 10–20 μm in diameter were placed into the sample chamber as pressure markers, following the relationship between *R*_1,2_-line shift and pressure.^49^ The hydrostaticity of the pressure-transmitting medium was monitored through the ruby R-line broadening, whose line width is known to slightly decrease with pressure in the hydrostatic range, and progressively broaden with pressure in the nonhydrostatic range.^33,50^ The relatively large size of the diamonds enabled us to load a significant amount of sample (0.1 mm^3^). However, it also limits the achievable pressure range to 5 GPa. In these experiments, we worked with AuNP colloids in EtOH as PTM, since it solidifies at about 3.5 GPa, thus enabling us to explore the effects of both hydrostatic and nonhydrostatic pressure on colloidal stability. SAXS images with 1 s exposure time were normalized and azimuthally integrated into curves using the local application Foxtrot, then further analyzed with the SASfit software,^51^ to test the geometries corresponding to each colloid and to explore different structure factors related to NP aggregation.

### Transmission Electron Microscopy

TEM images were obtained with a JEOL JEM-1400PLUS transmission electron microscope operating at an acceleration voltage of 120 kV. AuNP colloids were measured before and after pressure treatments. In the latter case, the sample was recovered from the pressure cavity of the gasket by transferring the colloidal mixtures onto a copper grid by touching the culet surface of the diamond anvil after pressure release. Although this method can accidentally drag some external AuNP off the hydrostatic cavity and onto the grid, compressed AuNPs can be readily identified by observing 1 out of 300 deformed NPs. This method allows us to explore the aggregation state, as well as size and shape of the compressed NPs.

## Supporting Information Available

(1) Lattice parameter *versus* nanoparticle volume relationship; (2) hydrostatic and nonhydrostatic pressure effects: reversibility; (3) the Williamson–Hall method; (4) uniaxial stress model; (5) SAXS *I*(*q*) pattern of *AR* = 3.4 AuNR solution in ethanol; (6) TEM images of AuNS before and after high-pressure treatment (PDF)
