# Supplementary material for: On the Stiffness of Gold at the Nanoscale
Source: ACS Nano. 2021 Oct 20;15(12):19128–37. doi: 10.1021/acsnano.1c06947 (PMC8717628; doi:10.1021/acsnano.1c06947)
Supplement: Supplementary file 1 — nn1c06947_si_001.pdf [file nn1c06947_si_001.pdf]

Supporting Information

On the Stiffness of Gold at the Nanoscale

Camino Martín-Sánchez[a], Ana Sánchez-Iglesias[b], José Antonio Barreda-Argüeso[a], Alain Polian[c,d], Jean-Paul Itié[c], Javier Pérez[c], Paul Mulvaney[e], Luis M. Liz-Marzán[b,f,g], Fernando Rodríguez[,a]*

[a] MALTA Consolider, DCITIMAC, Facultad de Ciencias, University of Cantabria, Av. Los Castros 48, Santander, 39005, Spain

[b] CIC biomaGUNE, Basque Research and Technology Alliance (BRTA), Paseo de Miramón 194, Donostia-San Sebastián, 20014, Spain

[c] Synchrotron SOLEIL, L'Orme des Merisiers St.Aubin, BP48, 91192 Gif-sur-Yvette, France

[d] IMPMC, Sorbonne Université and CNRS, 4 Place Jussieu, 75005 Paris, France

[e] ARC Centre of Excellence in Exciton Science, School of Chemistry, University of Melbourne, Victoria, 3010, Australia

[f] Ikerbasque, Basque Foundation for Science, Bilbao, 43018, Spain

[g] Centro de Investigación Biomédica en Red, Bioingeniería, Biomateriales y Nanomedicina (CIBER-BBN), Paseo de Miramón 194, Donostia-San Sebastián, 20014, Spain

Supporting Information content:

1) Lattice parameter *versus* nanoparticle volume relationship

2) Hydrostatic and non-hydrostatic pressure effects: reversibility

3) The Williamson-Hall method

4) Uniaxial stress model

5) SAXS $I(q)$ pattern of AR=3.4 AuNR solution in ethanol

6) TEM images of AuNS before and after high-pressure treatment

Lattice parameter *versus* nanoparticle volume relationship

Figure S1 shows the relative variation of the fcc lattice parameter of gold as a function the grain size from 2 nm to 2 µm at ambient conditions. Nanoparticle sizes smaller than 5 nm were determined by x-ray absorption fine structure at the Au K-edge,[1] whereas sizes bigger than 5 nm were obtained by high resolution x-ray diffraction (XRD) at synchrotron facilities (Ref. [2] and this work). Other earlier XRD data on AuNP were discarded due to insufficient resolution from XRD patterns,[3] strained NP samples[3] or lack size characterization,[4] all being unable for comparison studies due to large intrinsic errors. Data have been analysed using a semiempirical equation relating the relative variation of the lattice parameter in unstrained samples as a function of the NP volume. Beyond earlier models considering only surface effects,[5,6] the present model incorporates the effects of finite size of the particle in the binding energy, the smaller the size, the smaller the binding energy; the surface term due to the less short-range repulsive interaction by atoms at surface; and a third term due to increase of electron kinetic energy by quantum confinement. The curve corresponds to the fitting of data to this equation weighted by the accuracy of data. Note that there are three well distinguished regions associated to these terms, the surface and quantum confinement effects being evidenced in the smallest NP (1-5 nm). Within this model, the gold lattice parameter is within (0.01%) independent of the volume for grain sizes above about 80 nm.

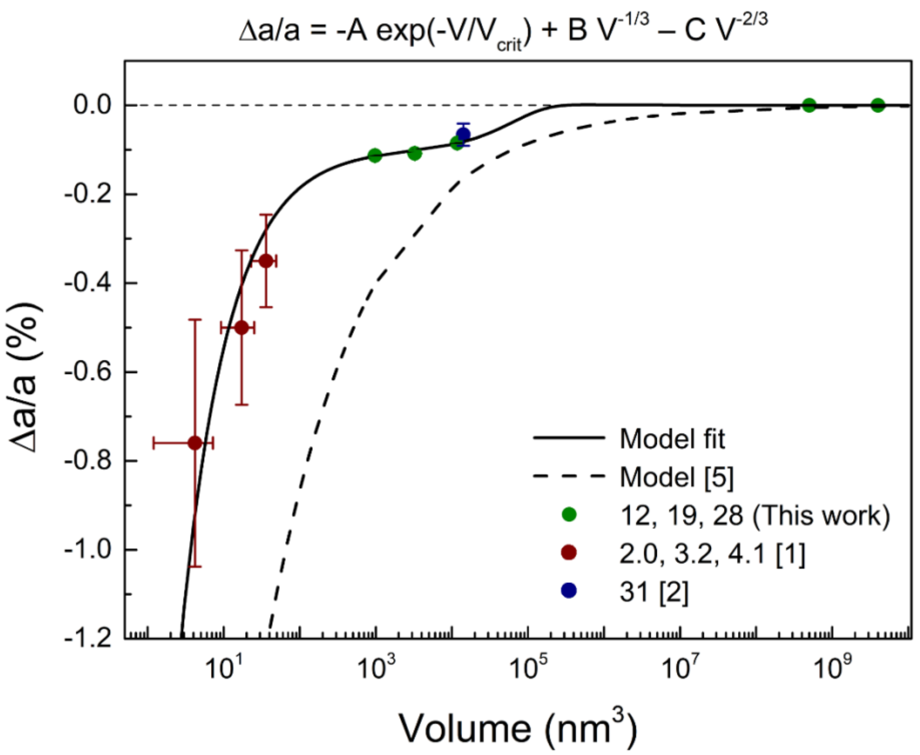

Figure S1. Variation of the relative fcc lattice parameter as a function of the volume of Au nanoparticles of different diameters: 12, 19 and 28 nm (green, this work), 2.0, 3.2, 4.1 nm (garnet, Ref. [1]) and 31 nm (blue, Ref. [2]). Bulk gold powder data with grain size of 1 and 2 µm have also been included (green, this work). Solid line corresponds to fit to the semiempirical equation indicated on figure top. Dashed line corresponds to predictions based on the Bond-Order_Length_Strength (BOLS) model reported elsewhere.[5]

Hydrostatic and non-hydrostatic reversibility

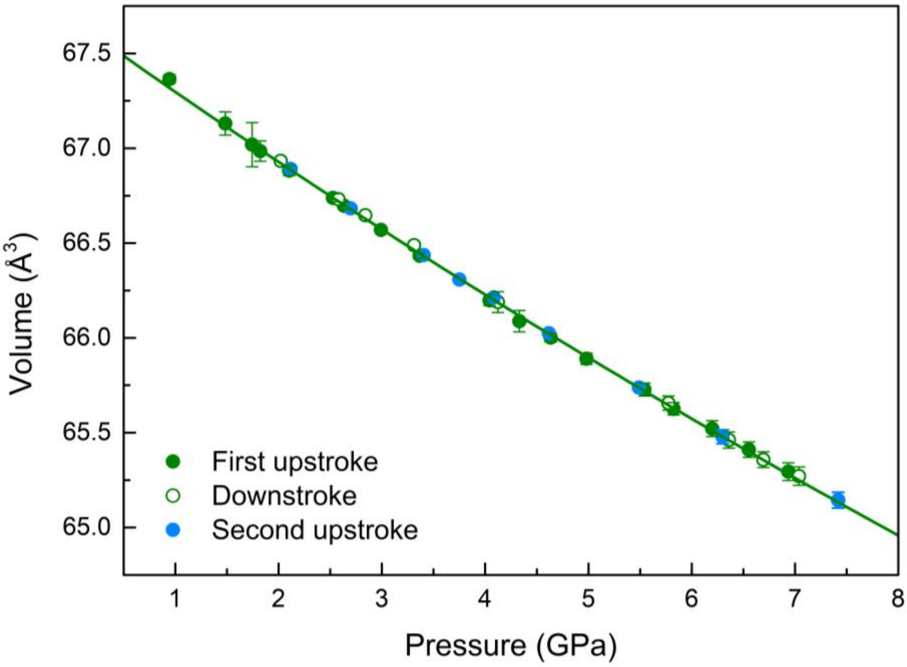

Figure S2. Pressure dependence of the 28.2 nm AuNS fcc cell volume in an upstroke-downstroke-upstroke cycle under hydrostatic conditions. The volume is within the experimental accuracy (0.03%) fully reversible.

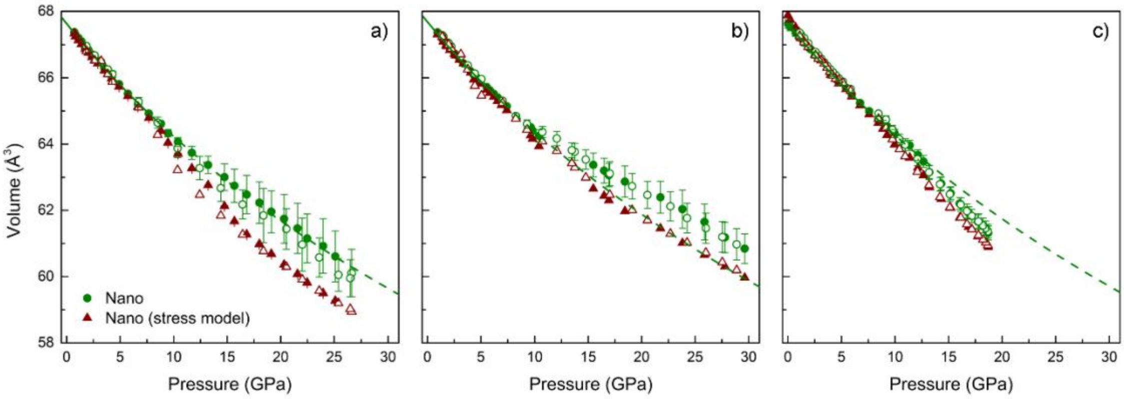

Figure S3. Pressure dependence of the fcc cell volume of a) 12 nm AuNS b) 28 nm AuNS and c) AR = 3.4 AuNR. Filled circles correspond to upstroke experimental data for (green) nano gold assuming a stress-free cubic system model; (garnet) nano gold considering stress following model reported elsewhere.[7] Empty circles correspond to the experimental points in downstroke. Solid lines correspond to fits to the Vinet EOS derived from *V*(*P*) data in the hydrostatic pressure range; dashed line corresponds to the extrapolated hydrostatic EOS. Error bars in volume are indicated or are smaller than the symbol.

The Williamson-Hall method

The Williamson-Hall method [8] allows us to calculate the crystallite size and the lattice strain from a diffraction pattern even when both are simultaneously present by assuming that the convolution of these effects can be approximated as a simple sum, obtaining:

$$B_r cos\theta = \frac{k\lambda}{L} + \eta sin\theta$$

where B_r is the peak broadening due to the combined effects of crystallite size L and lattice strain η, k being a constant known as shape factor. B_r can be obtained from the experimental spectra after subtracting the instrumental broadening using a gaussian approximation for a gaussian size distribution:

$$B_{exp}^2 = B_{inst}^2 + B_r^2$$

Here, B_{exp} is the measured full width at half maximum (FWHM) of the diffraction peak and B_{inst} is the instrumental broadening that has been determined from the XRD pattern of Au bulk. Similar results are found when using the XRD of a sample CeO_2 of high crystalline quality (U = 0.010156, V = -0.004079 y W = 0.002254) to account for the instrumental broadening: $B_r^2 = \mathrm{W} + \mathrm{V}\tan\theta + \mathrm{U}\tan^2\theta$.

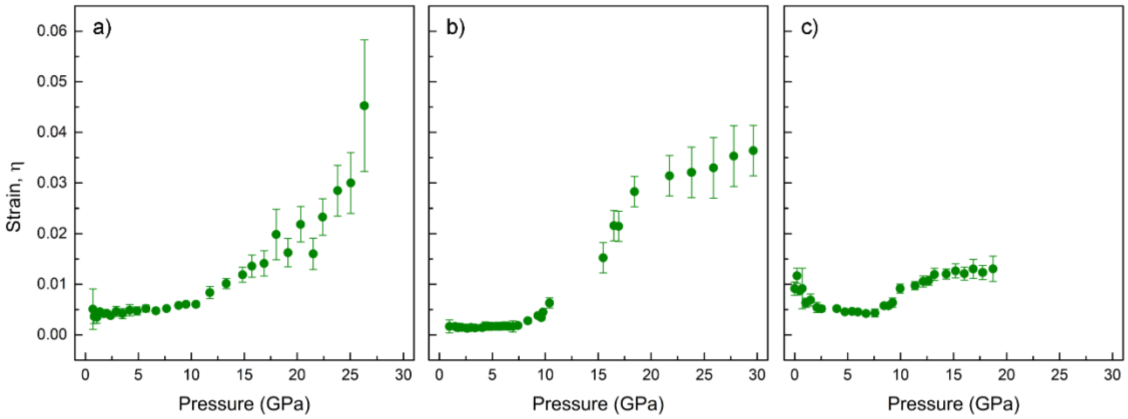

Figure S4. Pressure dependence of the Williamson-Hall strain of a) 12 nm AuNS b) 28 nm AuNS and c) AR = 3.4 AuNR colloids.

From the Williamson-Hall plots $B_r(hkl)$ *vs.* $\tan\theta_{hkl}$ we obtain an y-intercept of 0.0023(7), 0.0010(5) and 0.0008(6) for the 12.3 nm AuNS, 28.2 nm AuNS, and (AR = 3.4 AuNR) colloids, respectively. Considering the typical value of the shape factor $k = 0.89$ we obtain a crystallite size for the 12 and 28 nm AuNS of 14(4) and 33(16) nm, respectively. We obtain a reasonable value for the rod-like particles but with an overgrown uncertainty to be representative. Note that the strain in AuNR is significant lesser than in AuNS for the two investigated sizes, which reach values of about 0.03 at 30 GPa. However, it must be noted that this method is an approximation to describe the stress at the nanoparticle as each Bragg peak undergoes a slightly different broadening due to the lattice direction dependence of the Young modulus of gold.

Uniaxial stress model [7]

The uniaxial stress model provides an analytical equation relating the observed interplanar distances in axially stressed crystallites with respect to unstrained crystallites. For cubic crystallites under high pressure conditions, this model can be applied on the assumption that the stress acting in a particle can be separated into a hydrostatic term and a biaxial term yielding an effective uniaxial stress on the assumption that there is no strain perpendicular to the stress but along the stress. Considering the compression of a material inside a DAC which undergoes a homogeneous deformation, the total stress at the centre of the diamond anvil can be separated into the mean of the principal stresses σ_P (equivalent hydrostacic stress component) and the deviatoric stress component $D_{i,j}$ as:

$$\sigma_{i,j} = \begin{vmatrix} \sigma_{11} & 0 & 0 \\ 0 & \sigma_{22} & 0 \\ 0 & 0 & \sigma_{33} \end{vmatrix} = \begin{vmatrix} \sigma_P & 0 & 0 \\ 0 & \sigma_P & 0 \\ 0 & 0 & \sigma_P \end{vmatrix} + \begin{vmatrix} -t/3 & 0 & 0 \\ 0 & -t/3 & 0 \\ 0 & 0 & 2\,t/3 \end{vmatrix} = \sigma_P + D_{i,j} \quad (1)$$

where $t = (\sigma_{33} - \sigma_{11})$ is named as the uniaxial stress component (along the DAC axis).

The measured d-spacing $d_m(hkl)$ relates to the hydrostatic d-spacing $d_p(hkl)$ (under σ_P alone) following:

$$d_m(hkl) = d_p(hkl)[1 + (1 - 3cos^2\varphi)Q(hkl)] \quad (2)$$

where φ is the angle between the load axis and the normal to the diffraction plane ($\varphi = \pi/2$ for the parallel geometry used in most experiments as the present one) and $Q(hkl)$:

$$Q(hkl) = (t/3)\{\alpha[2G_R^X(hkl)]^{-1} + (1-\alpha)(2G_V)^{-1}\} \quad (3)$$

The subscripts R and V denote Reuss [9] and Voigt [10] approximations (stress and strain continuities, respectively). Our XRD patterns are satisfactorily accounted for under the Voigt approximation ($\alpha = 1$) with respect to a mixed Reuss-Voigt approximation. Finally, eq. (3) for a cubic system remains as:

$$Q(hkl) = t/3\,[S_{11} - S_{12} - 3S\Gamma(hkl)] \quad (4)$$

Where $\Gamma(hkl) = (h^2k^2 + k^2l^2 + l^2h^2)/(h^2 + k^2 + l^2)$ and $S = S_{11} - S_{12} - S_{44}/2$, being S_{ij} the single crystal elastic compliances.

Thus, the d-spacing expressions for the first reflections of the gold XRD patterns are:

$$d_m(111) = d_p(111)\left(1 + \frac{tS_{44}}{6}\right)$$

$$d_m(200) = d_p(200)\left[1 + \frac{t}{3}(S_{11} - S_{12})\right]$$

$$d_m(220) = d_p(220)\left\{1 + \frac{t}{3}\left[\frac{1}{4}(S_{11} - S_{12}) + \frac{3}{8}S_{44}\right]\right\}$$

$$d_m(311) = d_p(311)\left\{1 + \frac{t}{726}[128(S_{11} - S_{12}) + 57S_{44}]\right\}$$

$$d_m(222) = d_m(111)$$

$$d_m(400) = d_m(200)$$

Note that at variance with other diffraction geometries,[7] the parallel configuration with the x-ray wave vector **k** parallel to the DAC axis ($\varphi \approx \pi/2$), the measured interplanar distances are longer than the hydrostatic distances and therefore Bragg peaks appear shifted towards lower θ angles, depending on the *hkl* reflection, with respect to the hydrostatic interplanar distance for a compressive uniaxial stress. Therefore, in case of monodisperse distribution of randomly oriented nanoparticles, such shifts contain information on the uniaxial stress acting on the nanoparticle through the elastic compliances $S_{ij}(P)$ at a given pressure. Considering the elastic compliances of the bulk material, the present model provides the stress acting at the nanoparticle in a DAC whatever being the pressure transmitting medium.

Figure S5 shows the XRD pattern of 28.2 nm AuNS and AR = 3.4 AuNR at different pressures with the simulated pattern $I(2\theta)$ following an unstrained cubic NP model and the uniaxial stress model.

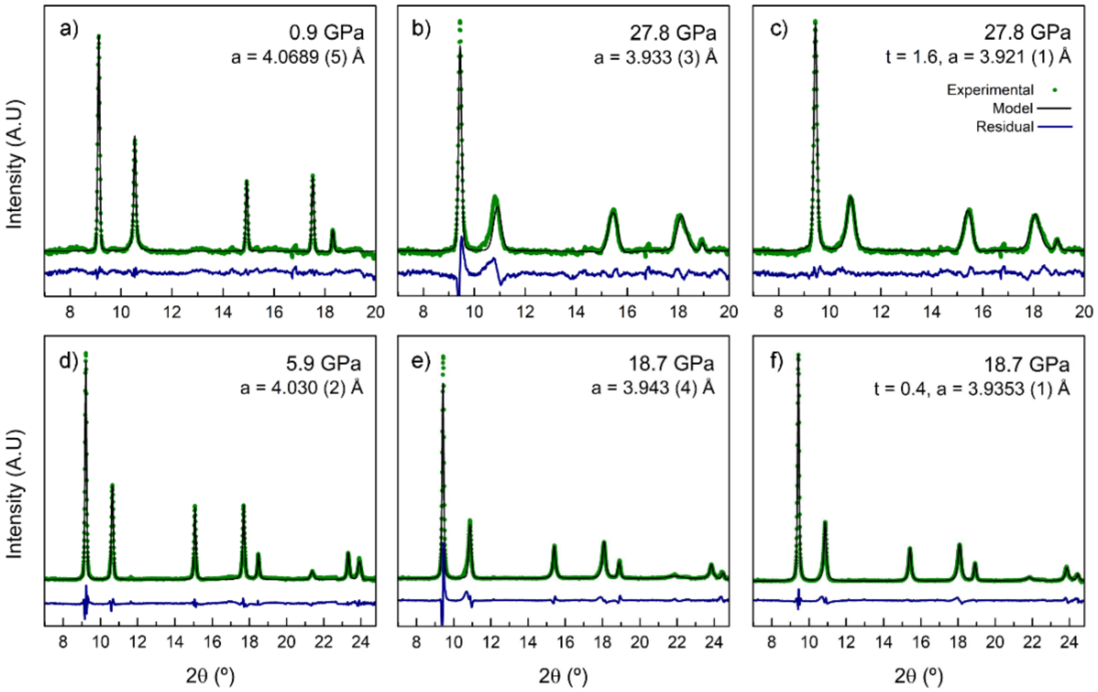

Figure S5. XRD patterns of 28 nm AuNS (upper panels) and AR = 3.4 AuNR (lower panels) for selected pressures in the hydrostatic and nonhydrostatic pressure ranges. XRD patterns a), b), d) and e) are modelled within a stress-free cubic system while patterns c) and f) are modelled considering the stress state of the system.[7] Note the abrupt deviation of the stress-free model in the non-hydrostatic region in XRD patterns b) and e).

Model limitations

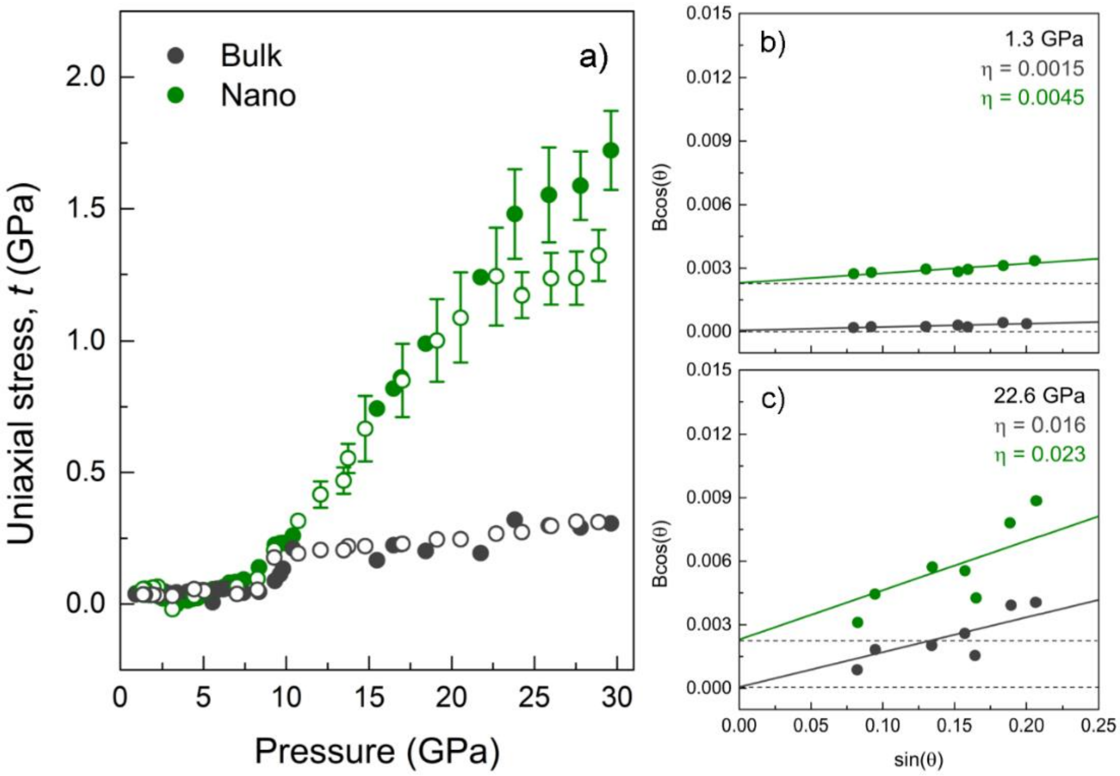

Figure S6. a) Uniaxial stress component $t = (\sigma_{11} - \sigma_{33})$ as a function of pressure, for 2 μm gold powder (grey) and 28 nm gold nanospheres (green). Empty circles correspond to the experimental points in downstroke. b, c) Williamson-Hall plots for selected pressures in the hydrostatic b) and nonhydrostatic c) pressure ranges. Note the different y-intercepts of the curves for the nanosized and bulk samples, derived from the different crystallite sizes and the strong increase in slope – *i.e.*, strain (η), under non-hydrostatic conditions.

Although the stress-model does not provide precise values of the lattice parameter, it does give information on the axial stress field acting on the NP, which in turn allows us to precisely identify the solidification pressure of the PTM and the increase in axial stress with pressure (Figure S6 a). The axial stress, which was derived from the Bragg peak shifts of the measured XRD patterns, was consistent with the NP strain derived from the Williamson-Hall (WH) plots [8] (Figure S6 b,c), relating XRD peak broadening to the combined effects of crystallite size and lattice strain (see Figure S4). Interestingly, while the uniaxial stress reaches values of 0.2 - 0.3 GPa for gold bulk in the studied pressure range, it reached 2 GPa at 30 GPa in AuNP. This means that the axial stress acting on the NP was enhanced by an order of magnitude with respect to that on bulk gold, even though both samples were measured under the same environmental conditions in the DAC. The WH-derived crystallite sizes for nano and bulk Au both coincided with their respective sizes measured in TEM, whereas the strain η increased upon solidification of the PTM, being η = 0.016 and 0.023 for bulk and nano Au at 22.6 GPa, respectively. The average stress associated with such strain $\eta = t/E$, with $E = 79$ GPa thus coincided with the average Young modulus of gold, yielding strain values between 1 and 2 GPa, in fair agreement with the stress derived from the Bragg peak shifts. The different broadening exhibited by

each peak in the W-H plots is noteworthy. The (111) and (222) Bragg peaks appear to be less broadened by stress than the (200) and (220) ones, due to the larger Young modulus along the <111> direction (E_{111} = 114GPa) than along <200> (E_{100} = 42GPa) and <220> (E_{110} = 81GPa) in bulk at ambient conditions.[11] These values change to 229, 73 and 149 GPa, respectively, at P = 22.6 GPa using the pressure dependence of the elastic compliances of bulk gold.[12,13] This distinct peak broadening is caused by the orientational dependence of the Young modulus, E_{hkl}, of cubic materials [14] yielding less broadened (*hhh*) peaks than (*h*00) or (*hh*0) peaks, since the corresponding FWHM is proportional to the strain $\eta_{hkl} = t/E_{hkl}$ for an applied uniaxial stress t. It must be noted that, although the effect of stress on the XRD pattern can be well accounted for using the stress model, it fails to provide precise $V(P)$ data in the non-hydrostatic region, since the hydrostatic volume derived from XRD underestimates the real volume in the parallel diffraction geometry of a DAC, as experimentally verified for several cubic systems.[7] This effect is even more delicate when dealing with NPs, since the stress is greatly amplified. Therefore, XRD data with non-hydrostaticity can yield erroneous $V(P)$ data, and hence disparity of EOS-derived bulk moduli in AuNPs, due to the higher sensitivity of nanoparticles to uniaxial stresses created by the solidified PTM.[2,15] This interesting finding explains the suitability of AuNSs to probe axial stresses in solidified PTM, as compared to bulk microparticles. A possible reason for this effect is the way solidified PTM transmits the axial stress through neighbouring microcrystallites, compressing the gold sample in microcrystalline powder or in AuNS. While the axial stress is homogeneously averaged over all PTM crystallites compressing the surface of macroscopic samples, in AuNS the number of crystallites compressing the nanoparticle is reduced, leading to larger fluctuations in the applied force. The effective force acting on the NP is difficult to predict *a priori*, since it depends on the microcrystallite structure (shape and size distributions) of the PTM, and on the way the force is transmitted to the NP. Consequently, this depends on both NP size and shape. The present XRD-based model provides a useful probe for experimentally determining the stress acting on individual NPs.

SAXS I(q) pattern of AR=3.4 AuNR solution in ethanol

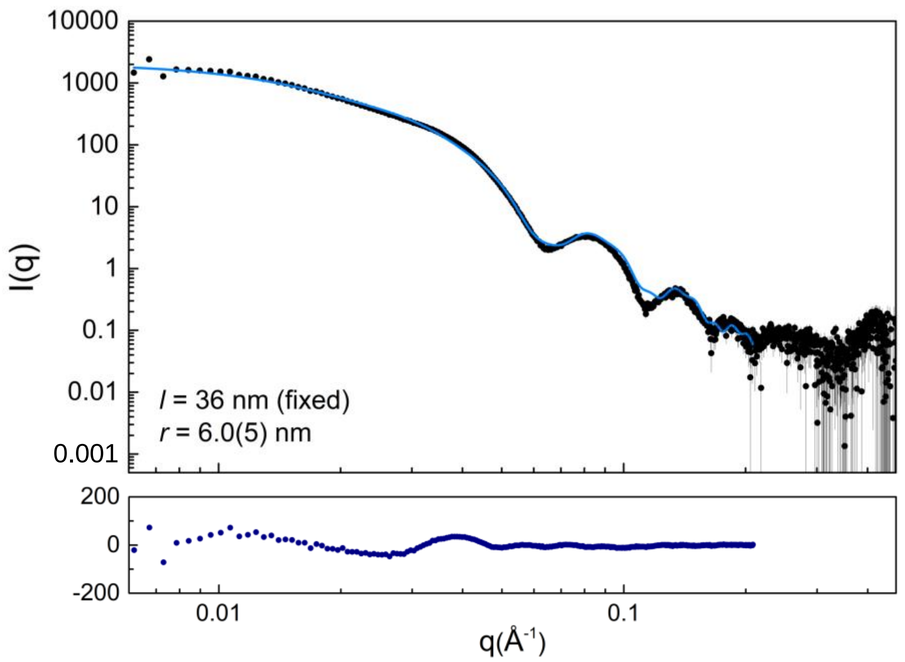

Figure S7. SAXS $I(q)$ pattern of AR = 3.4 AuNR solution in ethanol at ambient pressure. Filled circles correspond to experimental data and line represent the calculated $I(q)$ curve for monodisperse (individual) NP structure factor. Bottom curve shows the difference between experiment and calculations (fit residuum).

The $I(q)$ analysis confirms the stability of the colloids within the DAC, remaining monodisperse (individual particles) AuNR solutions. We fix the rod length to 36 nm and obtain a rod radius r = 6.0 nm with a standard deviation of 0.5 nm, a value fully consistent with the mean nanoparticle dimensions extracted by TEM, 36.1(6) $\times$ 10.7(4) nm^2.

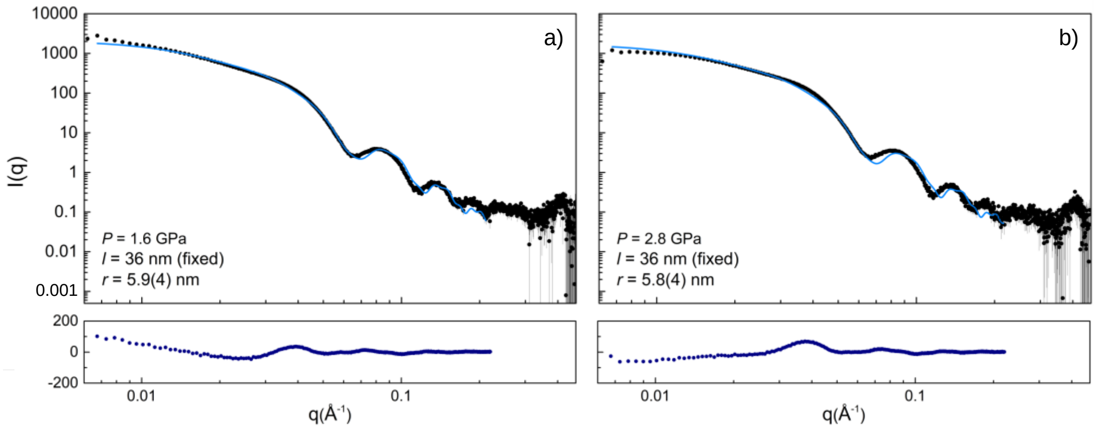

Figure S8. SAXS I(q) pattern of AR = 3.4 AuNR solution in ethanol for two selected pressures a) 1.6 GPa (hydrostatic) and 2.8 GPa (non-hydrostatic). Filled circles correspond to experimental data and line represent the calculated $I(q)$ curve for monodisperse (individual) NP structure factor. Bottom curve shows the difference between experiment and calculations (fit residuum).

TEM images of AuNS before and after high-pressure treatment

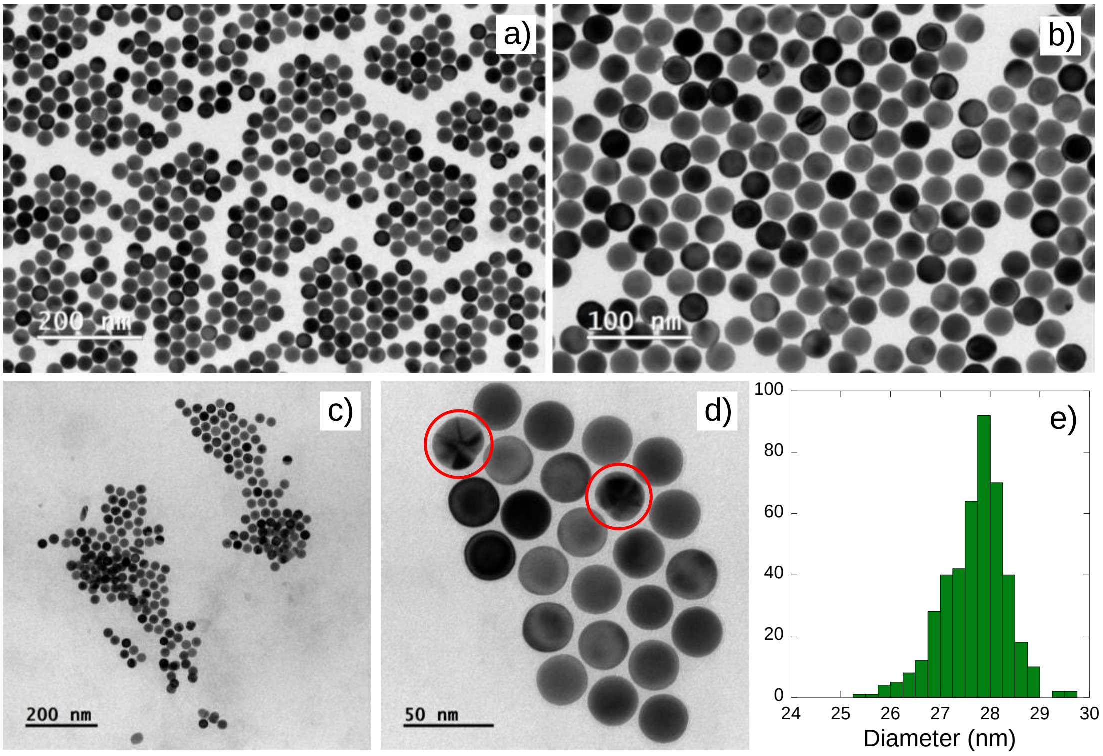

Figure S9. Representative TEM images of the a,b) initial MeOH-EtOH 4:1 28 nm AuNS colloid and c,d) recovered AuNS after being subjected to a high-pressure treatment of 31 GPa. Red circles indicate polycrystalline spheres slightly deformed under compression at 31 GPa. e) Diameter distribution of the recovered AuNS determined from TEM images.

Figure S9 shows representative TEM images of the MeOH-EtOH 4:1 28 nm AuNS colloid recovered after the pressure treatment up to 31 GPa (both hydrostatic 0-10 GPa and non-hydrostatic 10-31 GPa regimes). The initial spheres had a mean diameter of 27.8 $\pm$0.6 nm. After the pressure treatment the size distribution is found to be 27.7 $\pm$ 0.7 nm in diameter. According to this, we can conclude that there is no significant permanent increase in the size distribution of the nanoparticles. Despite being subjected to severe non-hydrostatic conditions, they maintain their original average dimensions. Interestingly, as illustrated in Figure S9d, single crystalline spheres are extremely stable under non-hydrostatic pressure whereas the polycrystalline nanospheres experience deformation.

On the other hand, the images reveal that there are some slightly deformed spheres. Images shows that AuNS reshaping is probably due to pressure-induced reshaping of individual nanoparticles due to non-hydrostatic effects. Note that the size/area of the reshaped nanoparticles is similar than the uncompressed nanoparticles. However, these deformed nanoparticles are not frequent, and represent a fraction of one out of 300 deformed AuNP.

REFERENCES

(1) Zanchet, D.; Tolentino, H.; Alves, M. M.; Alves, O. L.; Ugarte, D. Inter-Atomic Distance Contraction in Thiol-Passivated Gold Nanoparticles. *Chem. Phys. Lett.* **323**, 167-172 (2000).

(2) Gu, Q. F.; Krauss, G.; Steurer, W.; Gramm, F.; Cervellino, A. Unexpected High Stiffness of Ag and Au Nanoparticles. *Phys. Rev. Lett.* **100**, 045502 (2008).

(3) Mays, C. W.; Vermaak, J. S.; Kuhlmann-Wilsdorf, D. On Surface Stress and Surface Tension: II. Determination of the Surface Stress of Gold. *Surf. Sci.* **12**, 134-140 (1968).

(4) Martin, C. D.; Antao, S. M.; Chupas, P. J.; Lee, P. L.; Shastri, S. D.; Parise, J. B. Quantitative High-Pressure Pair Distribution Function Analysis of Nanocrystalline Gold. *App. Phys. Lett.* **86**, 061910 (2005).

(5) Sun, C. Q. Size Dependence of Nanostructures: Impact of Bond Order Deficiency. *Prog. Solid. State Ch.* **35**, 1-159 (2007).

(6) Qi, W. H.; Wang, M. P. Size and Shape Dependent Lattice Parameters of Metallic Nanoparticles. *J. Nanoparticle Res.* **7**, 51-57 (2005).

(7) Singh, A. K. X-Ray Diffraction from Solids under Nonhydrostatic Compression - Some Recent Studies. *J. Phys. Chem. Solids* **65**, 1589-1596 (2004).

(8) Williamson, G. K.; Hall, W. H. X-Ray Line Broadening from Filed Aluminium and Wolfram. *Acta Metall.* **1**, 22-31 (1953).

(9) Reuss, A.; Angew. Z. Berechnung der Fließgrenze von Mischkristallen auf Grund der Plastizitätsbedingung für Einkristalle. *Math. Mech.* **9,** 49-58 (1929)

(10) Voigt, W. *Lehrbuch der Kristalphysik* (Teubner, Liepzig, 1928).

(11) Martienssen, W.; Warlimont, H. *Springer Handbook of Condensed Matter and Materials Data,* 1, Springer Handbooks, Springer-Verlag Berlin Heidelberg, 2005.

(12) Daniels, W. B.; Smith, C. S. Pressure Derivatives of the Elastic Constants of Copper, Silver, and Gold to 10 000 Bars. *Phys. Rev.* **111**, 713 (1958).

(13) Golding, B.; Moss, S. C.; Averbach, B. L. Composition and Pressure Dependence of the Elastic Constants of Gold-Nickel Alloys. *Phys. Rev.* **158**, 637 (1967).

(14) Knowles, K. M. The Plane Strain Young's Modulus in Cubic Materials. *J. Elast.* **128**, 147-173 (2017).

(15) Hong, X.; Duffy, T. S.; Ehm, L.; Weidner, D. J. Pressure-Induced Stiffness of Au Nanoparticles to 71 GPa under Quasi-Hydrostatic Loading. *J. Phys. Condens. Matter* **27**, 485303 (2015).
